# Sarcopenia and Androgens: A Link between Pathology and Treatment

**DOI:** 10.3389/fendo.2014.00217

**Published:** 2014-12-18

**Authors:** Carla Basualto-Alarcón, Diego Varela, Javier Duran, Rodrigo Maass, Manuel Estrada

**Affiliations:** ^1^Programa de Anatomía y Biología del Desarrollo, Facultad de Medicina, Instituto de Ciencias Biomédicas, Universidad de Chile, Santiago, Chile; ^2^Programa de Fisiología y Biofísica, Facultad de Medicina, Instituto de Ciencias Biomédicas, Universidad de Chile, Santiago, Chile; ^3^Facultad de Medicina, Departamento de Morfofunción, Universidad Diego Portales, Santiago, Chile

**Keywords:** sarcopenia, androgen deficiency, elderly, skeletal muscle, testosterone

## Abstract

Sarcopenia, the age-related loss of skeletal muscle mass and function, is becoming more prevalent as the lifespan continues to increase in most populations. As sarcopenia is highly disabling, being associated with increased risk of dependence, falls, fractures, weakness, disability, and death, development of approaches to its prevention and treatment are required. Androgens are the main physiologic anabolic steroid hormones and normal testosterone levels are necessary for a range of developmental and biological processes, including maintenance of muscle mass. Testosterone concentrations decline as age increase, suggesting that low plasma testosterone levels can cause or accelerate muscle- and age-related diseases, as sarcopenia. Currently, there is increasing interest on the anabolic properties of testosterone for therapeutic use in muscle diseases including sarcopenia. However, the pathophysiological mechanisms underlying this muscle syndrome and its relationship with plasma level of androgens are not completely understood. This review discusses the recent findings regarding sarcopenia, the intrinsic, and extrinsic mechanisms involved in the onset and progression of this disease and the treatment approaches that have been developed based on testosterone deficiency and their implications.

## Introduction

In healthy young adults, the skeletal muscle mass comprises approximately 60% of total body mass. From age 40, the percentage begins to decline, reaching 40% at 70 years-old (1). This age-related decline in skeletal muscle mass and strength generation, the primary function of skeletal muscle mass, is known as sarcopenia (2, 3). This newly identified syndrome impacts both quality and quantity of life for both men and women, often leading to physical disabilities, gait abnormalities, and falls that cause loss of functional independence (4). Although sarcopenia is affecting more and more population worldwide, its pathophysiology remains unclear. Such lack of clarity can be attributed to difficulty in isolating the individual events responsible for alterations in skeletal muscle, most of which occur simultaneously, among the multiple age-associated changes and co-morbidities associated with advanced age. Indeed, most of the intrinsic as well as extrinsic (systemic) muscle changes that occur with age are believed to be involved in the development of sarcopenia (5, 6).

The difficulty in defining sarcopenia has created challenges in determining the best treatment for patients with this disease. Among the many different therapeutic approaches, including exercise, hormone, nutritional therapy, and/or a combination thereof, no one approach has emerged as superior in animal or human studies, and the great variability in the clinical outcomes of patients treated with these approaches has prevented identification of the most effective therapy (7, 8). One approach that has drawn recent attention is supplementation with androgens, hormones with anabolic properties whose levels naturally decline with age (9–12). Clinical studies of androgen supplementation in age-related diseases and muscle wasting are a focus of emerging interest (11). Skeletal muscle is a target tissue for anabolic steroids. Testosterone elicits significant muscular effects and abnormalities of plasma concentrations can cause muscle disease (13). High levels are associated with muscle hypertrophy, whereas low levels are epidemiologically associated with metabolic syndrome and diabetes, which negatively impact muscle functions. However, most evidence linked testosterone and skeletal muscle effects are observational. Studies targeted at establishing such effects at cellular level and their correlations with *in vivo* models, will broaden our understanding of the roles played by androgens on skeletal muscle function in elderly.

## Prevalence of Sarcopenia

The term *sarcopenia* was first proposed in 1989 by Irwin Rosenberg to describe a multifactorial syndrome that occurs with age and results in loss of skeletal muscle mass and function (3, 4). Prior to publication of the Report of the *European Working Group on Sarcopenia in Older People* (EWGSOP) in 2010, a variety of definitions of sarcopenia and inclusion criteria for patients with sarcopenia were used in epidemiological studies. In this report, the EWGSOP defined sarcopenia as a syndrome characterized by progressive and generalized loss of skeletal muscle mass and strength associated with physical disability, poor quality of life, and risk of death, and they also recommended criteria for diagnosis (14). Importantly, the recent development of these clear definitions and guidelines to diagnose and treat sarcopenia has significantly improved understanding of this relatively newly identified condition.

Although epidemiological research into sarcopenia has identified factors potentially associated with its prevalence, such as testosterone levels (15), most research has been cross-sectional rather than longitudinal, the approach that is necessary to increase our understanding. The prevalence of sarcopenia is partially dependent on the population studied, the measurement technique employed, and the operational definition used. Some authors report that sarcopenia affects both Hispanic and non-Hispanic white men and women, with prevalence ranging from 13.5 to 24% in individuals under 70 years, reaching 60% in individuals above 80 years. Also sarcopenia was significantly associated with several co-morbidities and lifestyle factors, including obesity, low income, current smoking, and lung disease (4). Regarding gender differences in muscle distribution, it has been observed that woman had up to 40% less muscle in the upper body but no more than 33% less mass in the lower body compared to man (16). At a transcriptional level, a marked sexual dimorphism was observed on the *biceps brachii* muscle. According to the authors, it appears that mainly age-induced changes at the transcriptional level in women, were responsible for increased expression levels of signaling pathways dealing with muscle dysfunction, inflammation, and mitochondrial dysfunction, thus leading to an alteration in “muscle quality” (17). On the contrary, regarding total muscle mass, age-related loss in total body muscle mass was greater in the male than female subjects, independent of change in stature, and can be clearly observed after approximately age 45. Also these results indicate that age-related loss of skeletal muscle mass is greater in the lower than upper body in both men and women (16). Clinical studies reveal that sarcopenia is a main cause of higher frailty, impairment, and loss of independence in elderly women than men (18). In addition, other cause contributing to sarcopenia is related to physical activity, which declines with age. Other indirect factors such as inflammation and oxidative stress also have been suggested to contribute to sarcopenia development. One of these factors that represent differences in men and women is the etiology of adipose tissue, which is a potent source of pro-inflammatory cytokines, suggesting that sex differences in total and regional adiposity can impact sarcopenia development.

Observation of a relationship between testosterone level and skeletal muscle anabolism has led to research evaluating age-related declines in testosterone levels. In a cross-sectional study, Feldman et al. (19) determined that total testosterone level declined approximately 0.8% per year and that both free and albumin-bound testosterone level declined approximately 2% per year after age 40, whereas sex hormone-binding globulin (SHBG) increased by 1.6% per year. In a subsequent longitudinal analysis, they determined that total testosterone declined by 1.6% per year and bioavailable testosterone declined by 2–3% per year. In a third study, they found that both testosterone level and the free testosterone index decreased progressively from the third to the ninth decades of life, specifically that total testosterone level decreased an average 0.110 nmol/L per year and the free testosterone index an average of 0.0049 nmol testosterone per nanomole SHBG per year, regardless of whether these variables were examined cross-sectional or longitudinally (19).

## Cellular and Molecular Mechanisms Underlying Sarcopenia

Although the pathophysiology of sarcopenia remains under investigation, two main groups of factors have been identified as responsible for its development: (1) intrinsic factors at the cellular level, such as mitochondrial malfunction and oxidative stress; (2) systemic factors, such as production of inflammatory cytokines, changes in hormone levels, and overall decrease in physical activity level. Loss of muscle mass and functionality related to sarcopenia cannot be completely explained by atrophy of muscle fibers. As such, the involvement of several atrophy-independent mechanisms in these conditions has been postulated, including decrease in myosin force and/or actin–myosin cross-bridge stability and defective excitation–contraction coupling (20, 21). From a descriptive/histological point of view, sarcopenia induces a change in the proportion of skeletal muscle fibers, inducing a shift from type II (fast) to type I (slow) fibers as well as preferential loss of type II fibers (22). This fiber-type change impacts not only the strength but also the power of particular muscles, and may be upstream of the signs of mobility limitation observed in sarcopenic patients (23).

### Extrinsic factors: Aging as a multisystem process

The aging process affects the physiology of many systems that work together to achieve the main function of the musculoskeletal system; namely, motion. Thus, age-related changes in these systems, especially those that affect neurological, inflammatory, and hormonal behavior may be directly related to sarcopenia.

#### Androgen levels as extrinsic factor for sarcopenia

In addition to a natural decrease in testosterone levels due to age, abnormal levels of plasma testosterone are observed in men suffering from late-onset hypogonadism, loss of testicular mass, and endocrine diseases involving low androgen production, accelerated testosterone metabolism or malfunctioning androgen receptor (24, 25). Currently it is recognized that low plasma testosterone is associated with metabolic syndrome, diabetes, and cardiovascular diseases (26); however, the impact of low plasma testosterone levels on skeletal muscle in elderly is still controversial. Because testosterone is the main physiological anabolic hormone, a decline in its plasma concentrations by age must be considered one of the causes for loss of muscle mass and an extrinsic factor for sarcopenia.

**Figure 1:**
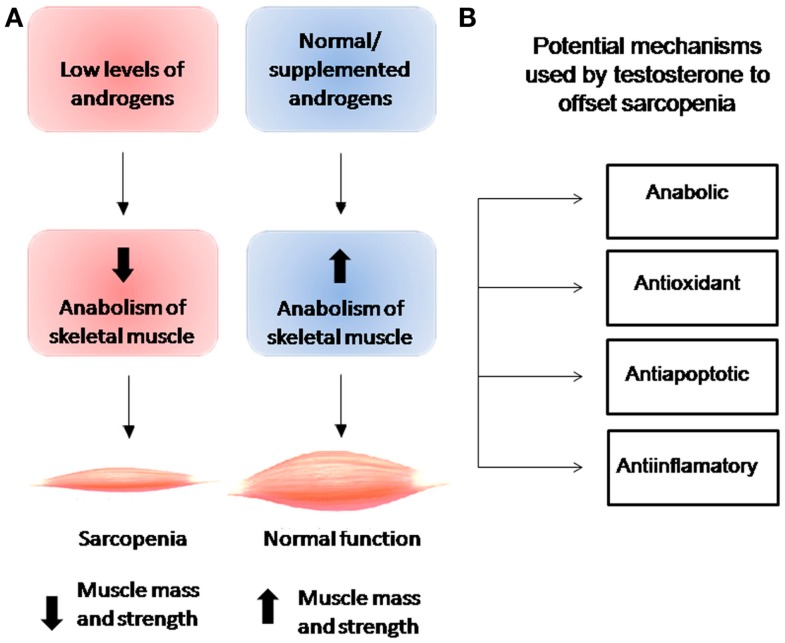
**(A)** Relationship between levels of androgens and anabolism of skeletal muscle and their effects on muscle mass and strength. **(B)** Cellular effects of testosterone on skeletal muscle.

Figure 1 shows the relationship between androgen levels and the development of sarcopenia. Androgen supplementation has been observed to exert anabolic actions that enhance muscle strength and increase muscle size clinically (27–31). Although the biological mechanisms underlying androgen action in skeletal muscle remain poorly understood, muscle mass has been observed to be regulated by the normal balance between synthesis and degradation of muscle proteins, a mechanism that is regulated by various systemic hormones. Levels of circulating testosterone, estrogens, IGF-1, vitamin D, and thyroid hormones have been observed to decrease with age (5, 32). While the association between testosterone and increase in skeletal muscle mass has been recognized for many years, it was not formally described until 1996, when Bhasin et al. demonstrated the anabolic effects of testosterone in a study of testosterone-treated vs. placebo-treated young men (9). While this study and subsequent studies provided evidence for anabolic properties of testosterone at all ages, none directly identified the mechanism underlying the relationship between testosterone levels and muscle mass with aging. In a cross-sectional study of 1,445 men, Krasnoff et al. (33) found no significant associations between either total testosterone or SHBG level and mobility, subjective health, or any physical performance measure, but did find an inverse association between free testosterone level and subjective health and a direct association between free testosterone level and usual walking speed and short physical performance battery score. In a subsequent longitudinal analysis, the authors found that free testosterone levels were also significantly associated with progression of mobility limitation (33). Specifically, they found that men with low free testosterone levels were 57% more likely to develop mobility limitation and 68% to experience increase in mobility limitation compared to men with normal testosterone levels. In support of this finding, Malmström et al. found that when the value is adjusted for height, bioavailable testosterone is responsible for 2.6% of the variance in appendicular skeletal muscle mass (ASM), which represents 75% of the total skeletal muscle mass (34).

Clinically, androgen supplementation has been observed to exert anabolic actions that enhance muscle strength and increase muscle size. While elevated plasma androgen concentrations are known to induce skeletal muscle hypertrophy (35), the cellular mechanisms by which testosterone increases skeletal muscle mass remain under investigation. Research to date indicates that testosterone stimulates protein synthesis by both a short-term mechanism-rapid activation of pre-existing components of the translational apparatus- and a long-term mechanism-increase in cell or tissue capacity at the protein synthesis level leading to increase in ribosome quantity (36). At the cellular level, Sinha-Hikim et al. observed that testosterone induces an increase in cross-sectional area (CSA) in type I and II muscle fibers and in myonuclear quantity, indicating that testosterone exerts more of a hypertrophic than a hyperplasic effect on skeletal muscle (30). At mechanistic level, have been observed that testosterone exerts a rapid non-genomic effect on skeletal muscle cells similar to that exerted by other steroid hormones such as estrogen, progesterone, vitamin D3, and aldosterone in different cellular types (37). In rat myotubes, a type of “immature” muscle fiber, our group recently observed rapid phosphorylation of Akt via phosphatidylinositol-3 kinase (PI3K) and subsequent phosphorylation of mammalian target of rapamycin (mTOR), which lies upstream of the critical translation regulator 40S ribosomal protein S6 kinase 1 (S6K1), to increase protein synthesis (38). mTOR represents a key controller of anabolic processes, particularly translation initiation and elongation that produce muscular protein. By activating mTOR, testosterone may induce phosphorylation of eIF4E-binding protein 1 (4E-BP1) and its release from eIF4E, a cap-binding factor that can be sequestered in inactive complexes by 4E-BP1, allowing for generation of initiation factor complexes (39). Moreover, testosterone-induced activation of the PI3K/Akt/mTOR/S6K1 axis combined with “classic” androgen-mediated action in rat myotubes has been observed to lead to hypertrophy, as evidenced by an increase in the CSA of testosterone-treated myotubes and levels of α-actin contractile protein (38). Other pathways have been hypothesized to underlie testosterone-induced hypertrophy. In a study of the cellular line L6, Wu et al. observed that testosterone treatment induced activation of mTOR, but more slowly (within 2 h) than had previous studies and in a manner not mediated by PI3K/Akt but rather MAPK (40). After confirming the anabolic potential of testosterone both *in vivo* and *in vitro*, White et al. described the activation of mTOR as dependent on the concentration of testosterone (41). For us, this point is critical to make comparisons between different studies and the effectiveness of therapy testosterone replacement, mainly because differences between the physiological effects of testosterone and changes in its plasma concentration, either low or high levels, may trigger different intracellular mechanisms for testosterone actions on skeletal muscle.

In summary, recent studies have found that testosterone activates multiple signal transduction pathways to induce hypertrophy, including the MEK-ERK and PI3K/Akt pathways; that the energy sensors mTOR and AMP-activated protein kinase (AMPK) control cell growth and metabolism in testosterone-induced skeletal and cardiac hypertrophy (38, 42); and that alterations in mTOR pathway in aging muscle decrease the kinase activity of mTOR and its downstream targets, decreasing anabolic activity in response to external stimuli, such as growth factor production, nutrient ingestion, and physical activity (43). In the emergent research into the early signals that mediate the activation/deactivation of these essential metabolic pathways, mTOR has been identified as a key element in the regulation of translation and cell growth; namely, as a “master regulator” in a network that detects the energy and nutritional status and genotoxic stress level of cells (44). Identification of the central role of testosterone in metabolism and its effects on mTOR pathway have led it to become a subject of intense interest in aging research and pharmacological treatment for sarcopenia.

#### Inflammatory cytokine production

Loss of skeletal muscle in sarcopenia may result from reduction in muscle protein synthesis and/or increased muscle protein breakdown, both of which are associated with inflammation. In this sense, other hypotheses regarding sarcopenia pathophysiology focus on the role of chronic low-grade inflammation, specifically that related to circulating levels of IL-6 and tumor necrosis factor-α (TNF-α). In a study of the relationship between muscle mass and strength in healthy individuals aged 70–79 years, Visser et al. found IL-6 levels of >1.80 pg/ml and TNF-α levels >3.20 pg/ml to be associated with smaller muscle area, lower appendicular muscle mass, and less strength, even after correcting for presence of inflammatory-associated diseases, height, and smoking (45). One mechanism hypothesized to underlie muscular atrophy in response to elevated TNF-α level is an increase in the ubiquitin-conjugating activity of the E2 protein UbcH2/E220K. In a study using cultured myotubes, Li et al. detected NF-κB mediated up-regulation of UbcH2 at the mRNA level after 90 min of TNF-α stimulation and at the protein level after 4 h of stimulation, as well as an increase in ubiquitin-conjugating activity after 4–6 h of stimulation to a level that remained elevated for at least 24 h (46). According to the authors, these findings could explain the mechanism whereby TNF-α may induce the loss of skeletal muscle proteins. In line with these findings, in a 5-year follow up study of older healthy individuals Schaap et al. observed a relationship between inflammatory marker (IL-6, TNF-α, and C-reactive protein) levels and a decline in muscle mass and strength, as well as consistent inverse association between levels of TNF-α and its soluble receptors and thigh muscle area and grip strength (47).

### Intrinsic factors: Changes at the skeletal muscle level

Intrinsic factors leading to alterations in skeletal muscle involve various intracellular signaling pathways, which in turn may activate intracellular modulators in a manner that has long-term cellular effects. The following sections describe several signaling pathways that may be involved in loss of strength due to atrophy of skeletal muscle fibers, a criterion for diagnosis of sarcopenia.

#### Mitochondrial malfunction and oxidative stress

Among the mechanisms hypothesized to underlie atrophic changes in sarcopenic muscles, mechanisms at the mitochondrial level have earned strong research support. In a cross-sectional study comparing active and sedentary elderly adults Safdar et al. (48) found that a sedentary lifestyle was associated with increased basal levels of markers of chronic inflammation (interleukin-6 and C-reactive protein levels), reduced level of markers of mitochondrial oxidative capacity (COX activity and levels of citrate synthase and PGC1-α levels), and deregulation of cellular redox status as superoxide dismutase (SOD) activity. All these changes, which are thought to render skeletal muscle fiber more prone to reactive oxygen species (ROS)-mediated toxicity and, consequently, to skeletal muscle fiber death, led the authors to suggest that mitochondria alterations and increased oxidative status in skeletal muscle are involved in sarcopenia development (48).

Oxidative damage and free radical production have also been associated with a decline in mitochondrial DNA content, protein synthesis, and activity, as well as in oxidative capacity and ATP production (49). In a comparison of aged (20 months-old) *Sod1*^−^*/*^−^ mice, a transgenic mouse model lacking the antioxidant enzyme CuZnSOD, and wild-type mice, Jang et al. observed greater ROS release (O_2_^−^ and H_2_O_2_) and oxidative damage and decreased mitochondrial bioenergetic functioning and ATP production in the *Sod1*^−^*/*^−^ mice (50). The authors hypothesized that the consequences of the mitochondrial dysfunction that they observed in the *Sod1*^−^*/*^−^ mice, which included elevated apoptotic potential; decrease in myonuclear number per millimeter of fiber length; and alterations in neuromuscular junction histology and contractility, also occur in the skeletal muscle of aging humans, and thus that mitochondrial dysfunction is an important factor in the pathophysiology of sarcopenia.

Recent *in vivo* and *in vitro* studies have highlighted the role of testosterone in preventing or reversing age-related skeletal muscle loss. In a recent animal study, a regimen of testosterone administration and simultaneous low-intensity physical training was found to improve skeletal muscle mitochondrial biogenesis and mitochondrial quality control in elderly male mice, suggesting the importance of maintaining proper testosterone levels for muscle metabolism (51). In various cellular models, mainly of prostate cancer cells, low levels of testosterone were found to be associated with oxidative stress induced by decreased antioxidant levels via decreased expression of antioxidant enzymes such as SOD, GSH-Px, and catalase (52). Whereas, testosterone deficiency has been observed to induce oxidative stress in cardiomyocytes, testosterone replacement therapy (TRT) has been found capable of suppressing oxidative stress mediated via the androgen receptor-independent pathway (52).

Skeletal muscle performance has been related to redox status. In soleus muscle of sedentary volunteers, Delgado et al. observed that administration of stanozolol, an anabolic steroid derivate, increased SOD, and glutathione reductase activities, but induced no change in other enzyme activities as catalase and glutathione peroxidase (53). A recent study shows that administration of dihydrotestosterone (DHT) to SOD1-G93A-mutated mice ameliorated muscle atrophy and increased body weight (54). As mutations in the human SOD1 gene are responsible for the development of amyotrophic lateral sclerosis (ALS), a late-onset neurodegenerative disease characterized by progressive loss of motoneurons, skeletal muscle weakness, atrophy, and paralysis, the authors suggested that androgen therapy may improve the quality of life for ALS patients. However, further study of antioxidant regulation by androgens in elderly humans with loss of skeletal muscle is required before extrapolation of these findings to sarcopenic patients.

#### Skeletal muscle apoptosis

Apoptosis, the process of programed cellular self-destruction without inflammation or damage to surrounding tissue, is highly related to the atrophic process that leads to sarcopenia (55, 56). Despite observation that this cellular response is driven by specific signaling pathways and ultimately executed through caspase-dependent and caspase-independent mechanisms (22), few studies have attempted to confirm the existence of these mechanisms in humans (57). Among the few that have, a cross-sectional study that compared apoptosis levels in the skeletal muscles of elderly and young adults observed a higher number of terminal deoxynucleotidyl transferase dUTP nick end labeling (TUNEL)-positive cells and decreased muscle strength in elderly but no significant differences in caspase-dependent pathway-related (caspase-3/7) activity or muscle fiber CSA between the elderly and young (56). In a study of the relationship between apoptotic activation in skeletal muscle and indices of muscle function and mass in the elderly Marzetti et al. found a correlation between biologic apoptosis markers and muscle mass and function indices in community-dwelling elderly men and women (58). Specifically, they found that caspase-dependent apoptotic signaling (caspase-8, cleaved caspase-3, cytosolic cytochrome *c*, and mitochondrial Bak activity) was correlated with percentage of muscle volume, whereas cleaved caspase-3, Bax, and Bak activity was correlated with gait speed. Although they found no significant differences regarding these apoptotic markers in subjects classified as either high functioning or low functioning according to whether their percentage of muscle volume was below or above the median value of the study sample, they observed higher levels of activated caspase-8 and cytosolic cytochrome *c* in the low-functioning subjects (57). These findings, in addition to evidence obtained from animal research, support the role of apoptosis as an important mechanism in the pathophysiology of sarcopenia.

Research has also indicated that testosterone has an anti-apoptotic effect in C2C12 muscle cells. Pronsato et al. found that treatment with testosterone at physiological concentrations inhibited apoptosis that had been induced by treatment with a high concentration of H_2_O_2_ (1 mM) (59). It also induced inactivation of BAD, inhibition of PARP cleavage, decrease in BAX levels, and exertion of a protective effect at the mitochondrial membrane potential level. In this line of evidence, testosterone administration prevented sarcopenia in a mouse model, which allow to hypothesize that testosterone treatment improves the regenerative potential of satellite cells and suppresses skeletal muscle cell apoptosis, the latter primarily by inhibiting activation of the JNK pathway and activating Akt (60). Further research has confirmed that testosterone supplementation exerts both beneficial and pathological effects on apoptotic process. Depletion of androgen below normal plasma levels has been found to increase kainate-induced neuronal loss (61) and induce apoptosis in the bladder wall of senile male rats (62), while elevated testosterone has been observed to induce apoptosis in a human neuroblastoma cell line (63). These findings indicate that the beneficial effects of androgen are obtained when androgen levels are within a specific (nanomolar) physiological range, the regulation of which is crucial for normal muscular functioning and a deviation from which in either direction could be deleterious to muscle health.

#### Muscle atrophy and Ca^2+^ signaling

The cellular processes underlying muscle atrophy result in both qualitative and quantitative alterations in muscle fiber cells and associated structures. Muscle atrophy leaves particular “signatures” in the profile of sarcopenic muscle proteins that can be observed, and muscle proteins are contained in different cellular compartments that can be analyzed. Since contractile proteins are critical for skeletal muscle function, analyzing changes in these proteins can assist in understanding the pathophysiology of sarcopenia. In a study of the synthesis of myosin heavy chain (MHC) protein over the lifespan and its correlation with muscle strength and hormone levels, Balagopal et al. (64) found that the rate of MHC synthesis begins decreasing at age 50 before plateauing at age 75, and observed a correlation between a decrease in the rate of MHC synthesis and muscle mass, strength measures and, interestingly, levels of anabolic hormones, including insulin-like growth factor I (IGF-I) and dehydroepiandrosterone sulfate (DHEAS) levels in both men and women and free testosterone level in men. As myosin represents about 25–30% of muscle protein, a decrease in its synthesis rate would be expected to impact muscle mass and strength (64). Other protein-specific changes in senescent skeletal muscle related to decreased muscle function involve α-actin, Ca-ATPase transporter, ryanodine receptor, and muscle-specific inositol phosphatase (MIP), a recently described protein related to Ca^2+^ homeostasis in skeletal muscle. In a recent study, Staunton et al. observed a lower level of thin filament α-actin protein in *vastus lateralis* muscle biopsy samples taken from aged subjects compared to those taken from middle-aged subjects (65). Utilizing mass spectrometry-based proteomic analysis, the authors were able to describe a “signature” of aged *vastus lateralis* skeletal muscle and detect changes in proteins involved in excitation–contraction coupling, metabolism, ion handling, and the cellular stress response. These findings led the authors to suggest that human muscle tissue undergoes a fiber-type shift from fast to slow contracting muscle tissue with age, an event that has been previously postulated and demonstrated in other sarcopenic models (65).

Concerning intracellular Ca^2+^ handling proteins in skeletal muscle, Ferrington et al. (66) described a decrease in the relative protein turnover of the sarcoplasmic reticulum proteins Ca-ATPase and the ryanodine receptor in aged rats. The authors hypothesized that alterations in the turnover rate of certain key Ca^2+^ handling proteins could account for the functional disturbances observed in skeletal muscle. Our research group has demonstrated that androgens are able to modulate intracellular Ca^2+^ homeostasis within seconds to minutes in different cell systems using a variety of mechanisms that vary considerably and depend on the cell type (67–69). In skeletal muscle, we observed that testosterone stimulation induces rapid (<1 min) oscillation in intracellular Ca^2+^ signals, which begin as Ca^2+^ transients initiated in the cytosol before being propagated as waves of Ca^2+^ in the cytoplasm and nucleus (68, 70, 71). This complex process of Ca^2+^ signaling, which depends on the interplay between IP_3_-sensitive stores and extracellular Ca^2+^ influx, is unaffected by the androgen-receptor antagonist cyproterone acetate, and apparently mediated by a pertussis toxin-sensitive G protein-coupled membrane receptor activated by testosterone 3-(O-carboxymethyl)oxime (T-BSA). Moreover, it has been suggested that a similar manner of conformational coupling between IP_3_ receptors and store-operated calcium entry (SOCE) is activated in skeletal myotubes during testosterone-induced Ca^2+^ oscillations (70). In this sense, age-related testosterone decrease certainly will affect skeletal muscle Ca^2+^ homeostasis.

As described by Shen (72) (MIP/MTMR14), a recently described protein, is responsible for sarcopenia pathophysiology by controlling intracellular phosphatidylinositol phosphate (PIP) levels via influencing SOCE and Ca^2+^ storage and release from the sarcoplasmic reticulum (72). Its importance is evidenced by observation of significant age-related decreases in levels of MIP mRNA, protein content, and activity in wild-type mice. Characteristic features of sarcopenia (diminished muscle mass, force, and power generation) also appear much earlier in MIP knock-out (MIPKO) mice than in their wild-type counterparts. Altered Ca^2+^ homeostasis has also been observed in both mature MIPKO and old wild-type mice (21). Moreover, muscle aging has been associated with compromised Ca^2+^ spark signaling and segregated intracellular Ca^2+^ release in sarcopenia (73). According to Romero-Suarez et al. (21), these findings all provide evidence of the important role of MIP in the physiology and pathology of muscle fiber.

#### Age-related depletion of muscle stem cells

The sarcopenic process is more pronounced in patients showing significant loss of muscle stem cells, particularly satellite cells. Several reports indicate that it is possible to isolate stem cells from adult skeletal muscle, but is important to distinguish between satellite cells and muscle-derived stem cells (MDSC). Embryonic stem cells and induced pluripotent stem cells are another cell source that can differentiate into muscle cells, but further work is needed to produce sufficient numbers of cells to generate contractile myofibers for therapeutic use (74, 75).

The satellite cells are mononucleated cells that lie under or embedded in the basal lamina of the myofiber, which demonstrate close relationship with the mature myofiber (75). The satellite cells are myogenic precursors capable of regenerating skeletal muscle and demonstrate self-renewal properties. During development and regeneration, quiescent satellite cells are activated and start proliferating, at this point they are called myogenic precursor cells or myoblasts (76). Once muscle satellite cells are activated to become myoblasts, they enter the proliferative stage and differentiate into myotubes by expression of MyoD, whereas, the secondary myogenic regulatory factors (MRF) as myogenin and MRF4 regulate terminal differentiations. Myofibers derived from satellite cells show characteristic skeletal muscle markers such as sarcomeric striations, MHC, MyoD, and desmin expression (77).

In the elderly, the number of satellite cells and their capacity of cellular regeneration decrease (78). In humans and mice, these quiescent cells are plentiful at birth but their number declines with age until 1–5% in adults (74). Different modulators regulate satellite cell functions in adults. Among them Alway et al. suggest that satellite cell function is affected by oxidative stress, which is elevated in aged muscles (79). During the differentiation of satellite cells to muscle a normal mitochondrial oxidative metabolism, with low production of ROS, is required to sustain skeletal muscle specification and function. However, aging is associated with excessive production of ROS by increase in mitochondrial damage contributing to impaired satellite cell function (79). Also, the decrease in satellite cells by age, has been attributed to the change in percentage of type I vs. type II muscle fibers, because satellite cells reside surrounding type II muscle fibers (46, 78).

Characterization of this satellite cells-derived skeletal muscle is determined at molecular, electrophysiological, and functional levels. At the molecular level, they show muscle-specific markers including transcription factors such as Pax7, Pax3, c-Met, M-cadherin, CD34, Syndecan-3, and calcitonin (80, 81). Recent experiments showed that, in contrast to cultured myoblasts, satellite cells freshly isolated or satellite cells derived from the transplantation of one intact myofibre, contribute robustly to muscle repair. However, because satellite cells are known to be heterogeneous, clonal analysis is required to demonstrate stem cell function.

There is consensus that the use of testosterone leads ultimately to regulate skeletal muscle mass by a net increase in protein synthesis over degradation. In addition for changes at the skeletal muscle level, it has been described that other cell types are involved in testosterone-induced muscle functions. Testosterone has direct effects on satellite cells, because they express the androgen receptor and in response to testosterone increase the satellite cell population. This cell proliferation is followed by a subsequent increase in the myonuclei number of the mature skeletal muscle, through the fusion of the satellite cells with pre-existing fibers resulting in muscle hypertrophy (12, 54, 82). Mesenchymal stem cells (MSCs) are a group of non-hematopoietic stem cells residing in bone marrow that can be used to treat a variety of degenerative diseases, including musculoskeletal diseases. The mechanism proposed to explain the effect of testosterone on fat-free mass, highlights the involvement of the androgen receptor in the commitment of an undifferentiated cell type into a myogenic cell line (83, 84).

## Therapeutic Approaches in Sarcopenia

Age-related frailty and sarcopenia have emerged as important public health concerns because of their negative impact on mobility, quality of life, and health care resources (85, 86). Nutrition and physical activity are two modifiable factors that can affect the development of sarcopenia. Improving either or both, nutrition and physical activity, may reduce the age-related low-grade chronic inflammation and/or activate the intrinsic anabolic pathways in skeletal muscle. While resistance exercise, which has assumed a prominent role in the treatment of sarcopenia, has been shown to improve skeletal muscle mass and strength in sarcopenic patients, it has not always been observed to improve functional parameters (87).

Androgen treatment has been observed to enhance skeletal muscle strength and size (30, 88, 89), but the biological mechanisms underlying androgen action in skeletal muscle are not completely known. In a review of six studies of TRT in elderly men, Borst reported that several studies found that testosterone treatment increased lean mass and decreased fat mass whereas one study observed increased strength (7).

### Anabolic interventions

Muscle plasticity and regeneration are key processes in a number of myopathies, including muscular dystrophy, neuromuscular disorders, and aging. Recent years have witnessed a growing interest in using anabolic interventions to counteract loss of muscle mass and function associated with age-related decreases in testosterone levels in men. Observation that low levels of testosterone are associated with decreases in fat-free mass, ASM, and strength in hypogonadal males compared to healthy controls has served as the basis to use TRT to treat hypogonadal men (90). With age, not only do testosterone levels decline progressively but SHBG levels also increase, further decreasing the amount of bioavailable testosterone. The prevalence of hypogonadism is approximately 20% in men over 60 years and can reach 50% in men over 80 years (31). In young hypogonadal men, TRT has been associated with increase in lean mass, muscle strength, and muscle protein synthesis and decrease in fat mass. However, in eugonadal men, changes in body composition following TRT have not always been followed by increase in muscle strength (91), leading to controversy regarding the ergogenic effect of TRT. In one study, TRT increased bone mass only in the group of patients with hypogonadism (89). In addition, several studies found that administration of supraphysiological doses of testosterone to hypogonadal patients resulted in outcomes similar to those obtained with resistance exercise (9).

Testosterone replacement therapy can be administered through several routes, including intramuscularly, transdermally, and orally (i.e., via testosterone undecanoate). Unfortunately, few studies of the safety of TRT using any of these routes, specifically its association with prostate cancer and cardiovascular disease in the elderly have been conducted. Moreover, the few studies that have been conducted were only observational or examined administration of only very high levels of testosterone. To date, TRT has been observed to induce and exacerbate sleep apnea, transient fluid retention, gynecomastia, increase red cell mass, and increase the size of both benign and malignant prostate tumors (31). TRT for hypogonadal patients has also been hypothesized to increase cardiovascular risk through its effect on lipid metabolism.

Many studies reporting an increase in muscle strength with TRT suffered from methodological problems, such as lack of a control group, lack of control for the effects of exercise, lack of control of the dose(s) of the hormones administered to maintain normal levels of circulating testosterone, or the inclusion of a very small number of patients. In a meta-analysis that pooled data from 19 randomized placebo-controlled trials, Calof et al. (28) reported that compared to placebo-treated men, testosterone-treated men were found 1.8 times more likely to experience prostate events, including prostate cancer, elevated prostate-specific antigen level, and prostate biopsy, but not at a level that differed significantly at the individual level. They also found that testosterone-treated men were, 3.67 times more likely to experience hematocrit over 50%, the most common testosterone-related adverse event, but did not experience cardiovascular events at a significantly higher rate (28).

In a randomized placebo-controlled trial of 44 men with late-onset hypogonadism aged 44–78 years, Marks et al. (92) found that 6 months of TRT normalized serum testosterone levels while only slightly and insignificantly increasing androgen levels in prostate tissue and volume, prostate-specific antigen, voiding symptoms, and affecting urinary flow, and did not alter prostate tissue composition, biomarkers of cell proliferation, gene expression, or cancer incidence or severity (92, 93).

Observation that levels of dehydroepiandrosterone (DHEA), a precursor of various sex steroids produced in the adrenal cortex, decline gradually with age from the third decade of life has motivated research examining whether DHEA supplementation can reverse the pathophysiological changes associated with aging. It is hypothesized that DHEA supplementation can increase muscle strength by increasing ratio of circulating testosterone to cortisol. In a study of men and women aged 50–65 years, (90) observed that supplementation with 100 mg of DHEA for 6 months increased lean body mass and decreased fat mass in both sexes, but increased muscle strength only moderately and only in men, and increased testosterone levels only in women. In a randomized placebo-controlled trial of 50 mg/day of DHEA supplementation for 1 year to men and women aged 60–80 years, (94) failed to reproduce Morales’s results or detect an increase in lean mass, as indicated by measurements of body potassium.

Oxandrolone, an androgenic steroid with powerful anabolic effects that can be administered orally, is resistant to hepatic metabolism and thus less hepatotoxic compared to other oral androgens. Moreover, its side effects, which include discrete elevations in transaminase levels and decreased high-density lipoprotein cholesterol levels, are mild and transient (95). Despite these advantages, no clinical studies of oxandrolone administration to elderly patients with sarcopenia have been conducted to date. However, previous studies of skeletal muscle wasting in cachexia associated with HIV infection, chronic neuromuscular conditions, and disease-related loss of muscle mass have indicated that oxandrolone increases protein synthesis in skeletal muscle, and is thus associated with increased physical activity capacity and level, increased energy and protein intake, reduced visceral and total fat mass, and improvement in nitrogen retention (96). Therefore, oxandrolone administration may be a therapeutic strategy in the treatment of sarcopenia in the elderly.

Supplementation with selective androgen-receptor modulators (SARMs) has emerged as a means of treating muscle and bone disorders, mainly because of the specificity of SARM action and the relatively few side effects of SARM treatment. Research using experimental models has demonstrated that administration of SARM S-4 exerts potent anabolic effects on skeletal muscle and bone and only minimal effects on the prostate (97). In a recent randomized, double-blind, placebo-controlled study of 170 women aged ≥65 with sarcopenia and moderate physical dysfunction found that 6 months of treatment with MK-0773 significantly improved physical performance measures (98). GTx-024 (enobosarm), a non-steroidal SARM that exerts tissue-selective anabolic effects in muscle and bone while sparing other androgenic tissue related to hair growth in women and prostate effects in men, has demonstrated promising pharmacologic effects in preclinical studies and favorable safety and pharmacokinetic profiles in phase I investigations. Thus GTx-024 supplementation resulted in dose-dependent improvement in total lean body mass and physical function and was well tolerated (99). Treatment with one or more of the numerous other SARMs currently under study may emerge as therapeutic alternatives to androgen agonist therapy. The intense research into pharmacological modulation of androgens and androgen intracellular signaling pathways may lead to the development of effective approaches to restoring and preventing the muscle loss observed in sarcopenia.

### Treatment alternatives and concerns

Because sarcopenia is a multifactorial disease, this should be treated using a variety of therapeutic approaches including diet, exercise, and pharmacology. Among current therapies, resistance exercise and androgen replacement therapy appear to be effective alternatives for sarcopenia because both modulate multiple tissues including musculoskeletal function. Therefore, in older men with sarcopenia a program of physical exercise together with TRT emerges as a promising therapeutic alternative. A great concern over the previous decade was that androgen supplementation increased risk of cardiovascular and prostate events. However, recent research has shown no association between androgen supplementation and increased cardiovascular risk (with several studies even identifying an association between low levels of testosterone and cardiovascular risk) nor between androgen supplementation and prostate disease. The development of “state-of-the-art” testosterone treatments has called for wide-scale longitudinal population studies to determine their profile. While other anabolic, such as DHEA, oxandrolone, and SARMs appear to be promising agents in sarcopenia treatment, further research is required before recommendations regarding their pharmacological use can be developed.

A decline in homeostatic and regenerative capacity occurs in aging, where a degenerative change in stem cells homeostasis has been postulated. Cell replacement therapy is a potential alternative treatment currently undergoing clinical trials (100). However, in elderly human muscle they are in extremely limited supply, hence there is a high demand for an alternative satellite cell source. There are a number of potential sources of muscle stem cells for cell replacement therapies such as bone marrow-derived stem cells, hematopoietic stem cells, and MSCs. However, there are significant controversies regarding both the efficiency and the reality of skeletal muscle differentiation by many of these stem cell types. Among these cell sources, satellite cells unquestionably undergo the most efficient musculogenesis. The actual focus in stem cell therapy, implicate to enhance satellite cell activity by environmental conditions and stem cell transplant into damaged tissues. Recently, it has been described that satellite cells can be transplanted into the muscle of mice, and they are able to proliferate. Moreover, these stem cells generate a massive proliferation during muscle injury (100). A recent *in vitro* study showed that treatment of 10T1/2 pluripotent mesenchymal cells with testosterone or DHT significantly increased the number of myogenic cells in a dose-dependent manner, while inhibiting adipogenic differentiation (101, 102). This study demonstrated that these effects are mediated through an AR-dependent mechanism, because an AR antagonist blocked the actions of testosterone or DHT. This hypothesis is also supported by the fact that in humans, CD34+ interstitial, mesenchymal cells are AR positive and expression of the AR is androgen dose-dependent (13, 102).

These data demonstrate that androgens can recruit stem cells into the myogenic lineage by committing them to myogenic precursor cells (101, 103). However, it is still not known whether androgen stimulation of myogenic commitment then gives rise to satellite cells, or directly contributes to muscle formation. Moreover, the physiological significance of the effects of androgens on stem cell commitment in contributing to muscle growth and regeneration is unclear. These preliminary studies clearly indicate that further investigation into this area is promissory and necessary.

## Conclusion

As an emerging syndrome with an increasing prevalence, sarcopenia has the potential to affect all aging adults. Therefore, we must consider that in older men with low testosterone levels and symptoms of androgen deficiency, hormone replacement therapy in combination with physical activity and proper nutrition will result in increased muscle mass and strength. However, data on the clinical effects of androgen replacement therapy to physiological ranges are not yet available. With the increasing aging of most of the world’s populations, research into this disabling disease, which not only decreases quality of life but also increases risk of mortality, is urgently required. Identification of the pathophysiological mechanisms underlying sarcopenia and the development of therapeutic approaches will improve the quality of life not only of the current elderly population, but all of us when we walked into our “golden years”.

### Highlights

- Sarcopenia is a decrease in skeletal muscle mass and function associated with age.
- Local and systemic factors as testosterone levels are associated with sarcopenia.
- No one of the several treatments for sarcopenia has been found clearly superior.
- Exercise, proper nutrition, and testosterone supplementation may treat sarcopenia.

## Conflict of Interest Statement

The authors declare that the research was conducted in the absence of any commercial or financial relationships that could be construed as a potential conflict of interest.
